# Exploring barriers, motivators and facilitators for physical activity and exercise in a UK South Asian community: a qualitative study

**DOI:** 10.1136/bmjopen-2024-097268

**Published:** 2025-02-19

**Authors:** Ashwini Deshmukh, Kanta Kumar, Jonathan Quinlan

**Affiliations:** 1 School of Sport, Exercise and Rehabilitation Sciences, University of Birmingham, Birmingham, UK; 2 School of Medicine and Health, University of Birmingham, Birmingham, UK; 3 Royal Wolverhampton NHS Trust, Wolverhampton, UK; 4 National Institute for Health and Care Research (NIHR), Birmingham Biomedical Research Centre, Birmingham, UK

**Keywords:** Exercise, Physical Fitness, PUBLIC HEALTH

## Abstract

**Abstract:**

**Objectives:**

This study aimed to elucidate motivators, barriers and facilitators of physical activity (PA) and exercise in a UK adult South Asian (SA) population. In addition, we sought to understand the sources of information regarding PA and the awareness of UK PA guidelines. Finally, the study aimed to explore public understanding of the utility of exercise for health outcomes and the role in disease prevention.

**Design:**

Explorative study using qualitative research methods including interviews and self-determination theory.

**Setting:**

Birmingham, UK.

**Participants:**

The study included 15 UK SA adults (8 male, 7 female) with a mean age of 53.1 years (SA defined as Indian, Pakistani, Bangladeshi or Sri Lankan).

**Results:**

We here found three key themes: (1) Engagement in PA and exercise, (2) Factors influencing PA and exercise (both barriers and facilitators) and (3) Accessibility to information. Participants showed a thorough understanding of PA; however, they lacked knowledge of strength-building exercise (ie, resistance exercise). This was particularly evident in SA women. Barriers to exercise typically focused around concepts of time, while facilitators centred on sufficient support. Knowledge of PA and exercise was typically obtained via social media, with only 2/15 aware of UK guidelines.

**Conclusions:**

While SA adults were aware of the importance of PA and its utility in health prevention, there was an evident lack of knowledge of guidelines and in particular the need for resistance exercise or other strength-building activities. We highlight a need to develop new routes to disseminate information within the SA population to increase knowledge and understanding of PA and resistance exercise for overall health.

**STRENGTHS AND LIMITATIONS OF THIS STUDY**

- This research was completed in accordance with the consolidated criteria (Consolidated criteria for Reporting Qualitative research) for reporting qualitative research.
- The interview guide was constructed through the use of patient and public involvement and refined through a public partner of South Asian heritage.
- We successfully recruited participants balanced for gender, major relevant religions and the multilingual researcher allowed for a wider societal representation.
- However, there may be an unbalanced socioeconomic representation, with participants largely well-educated and in professional job settings.

## Introduction

 Physical activity (PA) is a key regulator of overall health across the lifespan, reducing the onset and severity of many non-communicable diseases and as such, it is associated with reduced overall mortality.^1 2^ Similarly, muscular strength and muscle mass are also known to be vital for prolonging independence and extending health span.^3^,^5^ As such, declines in muscle health offer a key point of intervention with muscle weakness costing the National Health Service (NHS) approximately £2.5 billion annually.^6^ Thus the combination of daily PA and engagement in regular strengthening exercise (eg, resistance exercise) is paramount for health maintenance and prolonging quality of life. While PA and exercise are similar, for the purpose of this study, it is important to note the nuanced difference between the two entities. Specifically, PA refers to all physical movements completed in a day that require energy; whereas exercise (a subset of PA) refers to activities that are planned, structured and repetitive with the aim of improving physical fitness or performance.^7^ To this end, the WHO developed guidelines on ‘physical activity and sedentary behaviour’ which recommend adults engage in 75–150 min of vigorous-intensity or 150–300 min of moderate-intensity aerobic PA per week; in addition to two muscle-strengthening exercise sessions a week.^8^ Likewise, the UK’s chief medical officers’ PA guidelines, revised in 2019, follow a similar recommendation.^9^ Notably, both the WHO and UK guidelines place emphasis on regular resistance exercise activities.

Despite the evident health benefits of PA and resistance exercise, the Active Lives Adults Survey 2021 found that only 63% of UK adults (16 years+) were classified as being physically active and only 44% of adults engaged in resistance exercise.^10^ Engagement is lower in ethnic minority groups, with only 55% of Asian adults classified as active and 38% engaging in resistance exercise.^10^ Previous literature has shown that South Asian (SA) adults (ie, those from India, Bangladesh, Pakistan and Sri Lanka) engage in less objectively measured PA^11^,^14^ and have lower muscle mass and strength across the lifespan^15^,^17^ compared with age-matched UK white comparator groups. This is particularly concerning given the higher incidence of non-communicable disease in SA populations including cardiovascular disease and diabetes.^18 19^ It is also suggested that to obtain the same cardiometabolic risk score as white adults, SA adults may have to complete 10–15 min of additional exercise per day.^14^ As the SA population comprises approximately 9.3% of the total population (∼5.5 million people) in England and Wales,^20^ this reduced engagement in PA and exercise presents an opportunity for intervention to improve overall health outcomes and reduce comorbidities.

To cause measurable lifestyle change and improve health through appropriately designed interventions, it is imperative to understand what underpins existing lifestyle patterns in SA adults and elucidate any unique cultural barriers and/or motivators for PA and exercise. Previous research has indicated that relationships, knowledge, beliefs and environmental concerns (ie, access to facilities) often serve as barriers to PA and exercise in adult SA populations.^21^ While others have highlighted behavioural differences between first and second generation SA adults, with second generation engaging in higher levels of PA.^22^ Despite this initial insight, the fact remains that SA adults engage in lower levels of PA and exercise than other ethnic groups across the lifespan and thus further work is required to enable meaningful, culturally relevant lifestyle interventions. As such, the purpose of our study was threefold. First, to further elucidate motivators, barriers and facilitators of PA and exercise in a UK adult SA population. Second, to understand sources of information regarding PA and the awareness of UK PA guidelines. Third, to explore public understanding of the utility of exercise for health outcomes and the role in disease prevention. This data will be of benefit to future research projects exploring PA and exercise within a UK SA population with the aim of reducing health inequalities within the UK SA community.

## Materials and methods

This study began in January 2023 and was completed by December 2023. The study results are presented in accordance with the consolidated criteria (Consolidated criteria for Reporting Qualitative research) for reporting qualitative research.^23^ To gain insights into the public’s attitudes towards exercise and lifestyle adaptations, researchers in the field of health psychology have developed various theoretical frameworks. These frameworks aim to provide a comprehensive understanding of the factors influencing individuals’ decisions to either embrace or resist a healthy lifestyle. After thoughtful consideration, we chose to employ the self-determination theory (SDT) as our guiding framework.^24^ This choice stems from its ability to elucidate the motivation behind exercise as well as the barriers and facilitators associated with adopting and maintaining a healthy lifestyle. SDT is a motivational framework that has been widely applied across various aspects of life, including health. Health is an intrinsic goal for everyone, profoundly shaped by personal habits and lifestyle choices. Motivation, defined as the energy directed towards achieving a goal, plays a crucial role in influencing these choices and sustaining the necessary changes to maintain good health. According to SDT, three fundamental psychological needs underpin motivation: autonomy (the sense of willingly choosing and endorsing one’s actions), competence (the feeling of mastery and effectiveness in one’s activities) and relatedness (the need to feel connected to and supported by others). These needs collectively drive our capacity for sustained behavioural change and personal growth.^25^

### Participant recruitment

A total of 15 participants were recruited through online advertisements, via posters displayed in the local Birmingham SA community, for example, shops, places of worship and relevant community centres. Prospective participants contacted the lead researcher (AD) via provided details, and the study was explained in further detail. Prior to engaging in any study activities, all participants received the study information sheet, and written informed consent was obtained. A date was consequently organised to complete the interview. In total, 22 people declared an interest in the study, and the predominant reasons for exclusion were limited time availability, participants no longer wishing to partake or due to specific recruitment targets being met (eg, equal gender split). The recruitment of 15 participants was deemed sufficient as informed by previous literature^26^ and no further recruitment occurred. These sites of recruitment were used in order to reach directly into the local SA community and increase the socioeconomic range of potential participants. Recruitment occurred via convenience sampling methods due to the proximity to many large SA communities. All participants self-identified as individuals of SA heritage, including those of Indian, Pakistani, Bangladeshi and Sri Lankan descent. Demographic data, socioeconomic status and any underlying medical conditions for these participants are detailed in table 1.

**Table 1:** Demographic, socioeconomic and medical data of recruitment participants (n=15)

Demographics	
Age (years±SD)	53.1±12.4
Sex (m/f)	8/7
Religion	
Hindu (n)	6
Sikh (n)	5
Muslim (n)	2
Christian (n)	2
Immigration status	
Non-immigrant (n)	4
Years of stay in the UK, excluding non-immigrant (years±SD)	26.4±14.9
Highest level of education	
University (n)	9
College (n)	2
Secondary school (n)	4
Employment	
Professional (n)	6
Clerical (n)	2
Student (n)	1
Unemployed (n)	4
Retired (n)	2
Existing medical conditions	
Diabetes mellitus (n)	4
Plantar Fasciitis (n)	2
Hypertension (n)	1
Gout (n)	1
Hypothyroidism	1

Values listed as mean±SD.

### Data management

After participant consent was obtained, all participants were allocated a non-identifiable participant code (eg, PT001) for the remainder of the study. Signed consent forms which linked the individual to their unique code were stored in a locked cabinet in a keypad-accessed office. The participant code was used to store all collected data including demographics, socioeconomic status, medical conditions and the interview transcripts; all of which were stored electronically in password-protected files. Participants had the right to withdraw from the study at any point and remove their data from the study on request.

### Interview development and process

In partnership with our public partner, an interview guide was formulated, featuring a blend of questions framed within the SDT. This guide enabled the exploration of motivations, barriers and facilitators influencing SA individuals’ perspectives on PA and exercise (box 1). The questions were very specifically geared around the three fundamental psychological needs which underpin motivation: autonomy (the sense of willingly choosing and endorsing one’s actions), competence (the feeling of mastery and effectiveness in one’s activities) and relatedness (the need to feel connected to and supported by others).^25^

Box 1 **Interview guide employed during the study, split into three key sections**

**Physical activity (PA) versus exercise**

1. What do you understand by the term PA and/or physical work?
  - Do you think it is different from exercise? How?
2. Do you exercise?
3. On a day-to-day basis, what exercise do you do during a week?
  - Would you be able to exercise more?
4. How much hard physical work is required in your job/at home? Less, severe or moderate?

**Barriers and facilitators**

1. What motivates you to exercise regularly?
2. Are there any barriers or challenges that impact your ability to exercise?
3. Can you overcome these barriers? How?

**Access to information**

1. What are the sources from where you access information about exercise?
2. Are you aware of National Health Service Guidelines and Recommendations for PA and exercise?

Specific probes are listed underneath questions where appropriate.

The use of our public partner, who originated from a SA background, allowed us to tailor the questions meticulously to ensure the interviews generated meaningful and relevant data. Their personal experience as someone from the SA community played a crucial role in refining our approach. In addition, we built hypothetical vignettes to understand what actions would be taken in the following scenarios (table 2).

**Table 2:** The two vignettes provided to participants within the interviews

Vignette one	You start to notice that you are not as fit as you used to be, and activities such as walking up hills, climbing stairs and carrying shopping bags are becoming harder to manage. You decide it is time to start some regular exercise. What exercise would you do to help with the tasks above and how often would you do it?
Vignette two	You visit your general practitioner, and your recent blood tests have shown that you are now classified as ‘pre-diabetic’. The good news is that with some lifestyle changes (ie, exercise and diet) you can stop this progressing to a diabetes diagnosis. What measures will you take?

Semistructured interviews occurred online (n=13) with some occurring face-to-face in the School of Sport, Exercise and Rehabilitation Sciences at the University of Birmingham (n=2). English was the predominant language used during interviews (n=11) with some occurring in Hindi (n=4). Importantly, all interviews occurred in a one-to-one fashion within an enclosed room. The interviews were all completed by a female research associate, AD, who had close to 3 years of previous experience in conducting interviews. Interview transcription was consequently completed by an independent professional company, with interviews completed in Hindi translated into English. Using the Mapi Institute guidelines,^27^ the interviews were directly translated from Hindi into English from an audio recording. We did not complete any specific back translation as the translated script was read by the research associate who was fluent in Hindi, and these were checked against the audio recording for accuracy.

### Thematic analysis

To gain a comprehensive understanding of the interview content and delve deeper into the research topic, we employed a qualitative thematic analysis framework using an approach informed by Braun and Clarke.^28 29^ Categories emerged organically from the interview text, and the analysis involved interpreting the underlying meaning of the content. Each interview served as a unit of analysis, and rigorous discussions were conducted by the research team to ensure the reliability of the findings. The analysis began with multiple readings of the interview text by the research associate to establish a shared sense of the narrative that was then validated by the wider research team. Meaning units related to the study’s aims were identified, and a spreadsheet was created to capture initial broad coding. These meaning units were coded and grouped into categories which were then interpreted and abstracted into themes. The analysis process involved continuous iteration among meaning units, codes, categories and themes. The research team independently reviewed the coding, and the study results were derived from ongoing team discussions, reflections and agreements, all guided by consensus and aligned with the study’s aims. A meeting took place with our public partner to gain their agreement on the overall merging themes, and any discrepancies were corrected.

### Patient and public involvement

Our public partner assisted in the design of the interview guide. He has been collaborating with the research team for 8 years assisting in numerous studies of a similar design with a background in long-term chronic disease. In this case, through a series of meetings, we discussed the interview topic guide and dialogue around SDT (motivation). The interview topic was also piloted, and this provided direct feedback which was incorporated, for example, certain questions were asked in an alternative way. Further, our public partner was involved with the analysis and interpretation of transcripts and agreed on the conclusions that the researchers reached from collected data. Participant consent was obtained to allow the public partners to review interview text; however, all identifiable data was removed from transcripts and only identified by unique study code. All public partners were provided remuneration for their time and contribution.

## Results

The analysis resulted in three overarching themes: (1) The ability to engage in PA and exercise, (2) Barriers and facilitators to enhance exercise engagement and (3) Exercise-related information and knowledge. Alongside these themes, we incorporated two vignettes to delve deeper into participants’ perspectives on the relationship between exercise and disease.

### The ability to engage in PA and exercise

Participants varied their views on describing exercise engagement compared with being physically active (Quote 1, 2). There was greater emphasis on describing PA and being active through regular walking, housework, yoga, while some mentioned the use of the gym. There was little focus on resistance exercise but instead on keeping muscles ‘active’. However, there were strong views on integrating PA into daily life, and it was widely perceived as valuable and desirable, despite challenges in its actual execution (Quote 1, 2, 3).

Q1: For me, it’s anything that doesn’t involve just sitting down or standing still, so it could be walking, jogging, swimming, going to the gym.

Q2: The physical work is something which you actually have to get up and go about doing it rather than sitting in one place, so it could be going for a job, going for a walk, going to the gym, using the equipment in the house. If you have any and things like that or even like some rigorous house work as well. So it could be cleaning and stuff like that, but it has to be obviously quite rigorous for it to be counted as physical activity, so this is all the activities I can think of.

Q3: I tend to regard those exercises so when I’m doing the ironing at home, I’m standing up and moving around as I’m moving clothes back and forth on the iron, so that to me would mean I’m physically active, but I wouldn’t say it’s exercising when I’m doing the ironing.

Participants expressed a determination to be physically active, even in the face of pain or other obstacles, demonstrating a keen desire to incorporate more exercise into their daily routines (Quote 4). PA was considered a fundamental necessity, with participants displaying a nuanced understanding of its importance, although the duration of daily engagement varied among them. Moreover, PA was deemed essential for enhancing overall quality of life and seen as a prerequisite for other activities (Quote 5, 6).

Q4: I know if I sit for too long, I will get worse. Yes, I am in pain at times with my joints, but I think keeping moving is best for me.

Q5: I might go jogging once or twice a week. And depending on work, if I have to drive to work, I can’t. But if I can, I’d rather walk or get the bus to work and then I might. I’d like to try and do even on the days when I’m not exercising. I like to try and do between 8 and 10,000 steps a day, so just like do a lot of walking.

Q6: Keeping active has become so important now and it benefits our health problems too. Our jobs are all sitting down and I don’t see any other way than being active.

Participants anticipated both short-term and long-term health benefits from regular exercise, viewing it as a preventive measure against diseases and a potential alleviator of pain. Mental well-being was another considerable motivator, as participants believed that exercise contributed to mental relaxation and focus (Quote 7, 8). The invigorating aspects of exercise were also highlighted as compelling reasons for maintaining an active lifestyle. Cultural aspects of exercise received limited attention in the discussion in this section.

Q7: I’d certainly be, you know, do more physical activity. I don’t mind it. I’d rather be physically active than me sitting down.

Q8: Keeping active and fit has more important than ever. We all travel in cars and work at the desk and exercise is important for the mind and body. I find yoga and walking very useful to keep me well and relaxed.

### Barriers and facilitators to enhance exercise engagement

While weather was discussed in some interviews as both a facilitator and a barrier, other factors took precedence. Participants’ lifestyles, their work and the demands on their time were a great barrier (Quote 9, 10). Participants in full-time work and in professional jobs expressed more concern about the lack of flexibility in their working diaries. Other barriers such as lack of motivation and a desire for group support, particularly through walking groups.

Q9: You see my work is shift patterns and trying to work out a way to exercise is really challenging. I wish I had more time in the day to be active, but I think everyone feels work life and trying to be active is difficult.

Q10: Yes, although work is a hinderance, but we just have to find ways to be more active. The weather isn’t great in the winter but what can we do living in this country.

There was a concern that due to cultural events in the Asian community, setting timings for activity or exercise also acted as a barrier (Quote 11, 12). The most frequently cited facilitators were social support, enjoyment of PA, progress recognition, incorporating activity into daily routines and scheduling time in their working diaries. Other commonly mentioned barriers encompassed competing commitments, for example, housework, family life and illnesses, mental health issues, low energy levels and negative thoughts (Quote 13, 14).

Q11: Sometimes I feel I can spend more time at the weekends to exercise but in our circle family wedding or parties or someone could be visiting you just makes it really hard to plan a proper timetable…I think…

Q12: We do have lots going on in the Asian community events and all and lots of food too.

Q13: On the Saturday, I will clean the flat so I can do 8000 steps. Sundays I don’t spend as much time cleaning, but I might spend some time doing some ironing on a Sunday, you see? So I tend to do my washing twice a week, so I do 2 lots of ironing the week, so I’ll do that standing up. Watching television, and even then I’m moving around.

Q14: Well, it’s a lot of things. It’s health related. I’m in my mid 50s now. And as you get older, you have to build my active life. I also have diabetes and so I have to think about this too. Sometimes I do get down with my illness and don’t want to anything but then I have to tell myself I have to.

Some participants found increased activity easier with sufficient social support, family support or commitment to a goal. Lack of support meant less activity or exercise (Quote 15). We inquired with participants about factors that could improve their motivation or support them in engaging in more PA. Here, participants mentioned ideas they had observed in other countries; laughter parks, buddying schemes, time to be built in working contracts for exercise (Quote 16, 17, 18).

Q15: I have lots of house chores to do when I get home from work and at the weekends it’s the same. I should ask for more help from the family really.

Q16: I think a big thing would be to make sure it wasn’t too expensive [gym membership]. I pay £25.00 a month, but in the past I’ve come across charges of £50 or £70 a month.

Q17: They tended to be male, I think more South Asian women would probably use the gym I’m using now than used the previous gym, but I can only go for while you see. I don’t know. I mean I do see South Asian people using the gym where I go.

Q18: In other hot countries we see more effort being made for people to exercise like I have seen laughter parks full of people. Can you imagine to have this here? I think there does need to be more done on getting people together to exercise. A group or something for people to belong to.

Participants in the study highlighted the need for culturally sensitive fitness options, suggesting that separate gyms for females could address the discomfort some may feel in mixed-sex gyms (Quote 19). Additionally, while not the focus of the interview, discussions centred on the importance of educating the SA community about calorie intake and dispelling misconceptions about diet (Quote 20, 21).

Q19: I think if the Asian women had more gyms to go to they would, some are shy. Some don’t know what to do so having a buddy scheme as well.

Q20: Our Asian community need to have more understanding on food. I say this because I bet people don’t know much about calorie intake. The older generation don’t make the links to what they eat all day and what needs to be burnt off I guess that’s the same for a lot of mid aged people too. I think there needs to be more on this and the way it would make sense to the Asians. The community can help in temples talks and social media.

Q21: I don’t know if GPs tell people about calories intake in the way people would understand there needs to be more information on this I think and then you can understand the exercise.

There was a consensus that while the community is aware of the risks associated with poor dietary habits, there is a lack of motivation to change and incorporate regular exercise as part of a lifestyle change. The participants emphasised the necessity for more education on different types of exercises and their health benefits, particularly expressing a limited understanding of muscle strength and resistance exercise (eg, weight training) (Quote 22, 23). This knowledge may come from regularly attending a gym, and as such, if people are not part of the gym, then there is a danger of missed opportunity to educate on different forms of exercise. Concerns were raised about the affordability of gym memberships, with participants noting that individuals in lower-income jobs may struggle to access membership-based gyms. Therefore, individuals suggested that city councils and sport organisations need to take more action and lead initiatives to accommodate people on the lower-income jobs (Quote 24, 25).

Q22: See go to the gym to do more cardio but the I haven’t been told about muscle building. There needs to be more on this in my view.

Q23: I don’t think Asians understand the muscle build bit. General exercising – yes there is an understanding.

Q24: Some people don’t earn that much to pay for the gym. So should the council have more places for people to exercise?

Q25: I see the workplace could do more. I know there are places in other countries, where employers let the workforce look after their wellbeing a lot more than we do here. I think it should be in our contracts to be fit and health scheme.

### Exercise-related information and knowledge

When exploring the sources of exercise-related information among participants, a range of channels surfaced. Participants who spoke languages other than English primarily obtained exercise information from SA social media, particularly through platforms like YouTube. These sources also offered insights into aspects of healthy living, such as managing diabetes or improving blood pressure. Some individuals turned to Google for information, while others relied on sources like gym trainers, family, friends and healthcare professionals, including general practitioners. Surprisingly, only two participants, both working in the healthcare system, were familiar with the NHS guidelines on exercise. Overall, information about exercise tended to circulate within the community and on familiar personal platforms (Quote 26, 27, 28).

Q26: My daughter is my main person to tell me where to watch the videos about exercise. She shows my all of these on YouTube.

Q27: I get my information from the social media like if see any yoga class in the area or sometimes I get the information from my temple too.

Q28: I would say I get the information from my GP mainly. I know there are lots other places too but that’s a good way of getting information.

### Action to counter health threats (vignettes)

We employed two vignettes to assess participants’ knowledge regarding the recognition of health threats and the actions they would take (table 2). The first vignette aimed to determine participants’ ability to identify resistance exercise. While participants responded well by expressing intentions to increase overall exercise, incorporate more cardiovascular exercise, include hill walks, build stamina and adjust their diet; there was a noticeable lack of emphasis on resistance exercise. This underscores a knowledge gap in different types of exercise, suggesting that addressing a health threat may not yield optimal results without comprehensive exercise strategies (Quote 29, 30).

Q29: I would start more hill exercising and make sure I do cardio.

Q30: I would look to see if I can increase my times for exercise and ask the GP to help with the plan I would do more walking and eat less and do more cardio.

The second vignette focused on individuals being informed of pre-diabetes, with a specific interest in participants' awareness of NHS PA guidelines. While participants proposed innovative actions to reduce the risk of becoming diabetic, there was limited mention of adhering to NHS guidelines (Quote 31, 32). However, participants did express willingness to follow advice from healthcare professionals. Despite variations, participants generally understood the need for behaviour change in response to health threats, whether through dietary adjustments or increased exercise levels. Notably, only a few mentioned the NHS healthy plate, suggesting a potential need for greater cultural reference in health guidelines. (Quote 33)

Q31: Everything is on social media, I don’t think anyone will know the official guidelines to be honest.

Q32: I have never heard of guidelines I just look at the TV to see what I need to do. I have diabetes and I also have information from my hospital as well.

Q33: I only know about these guidelines because I work at the hospital otherwise, I don’t think people in the Asian community will know to be honest. May be there should be efforts to try to show these again the way it makes sense for the people in different cultures and ethnic groups.

## Discussion

Through employing the SDT framework principles, we here report key components of the SDT theory including autonomy, competence and relatedness. Our data reveal a clear appreciation for the need for proactive lifestyle measures to address emerging health threats such as diabetes and/or muscle weakness and the utility for overall health maintenance. Several motivators and facilitators for PA were identified, and we observed that engagement in resistance exercise may be linked to a lack of specific knowledge.

The forms of PA or exercise typically discussed were low-intensity options such as walking and yoga with a focus on cardiovascular exercise; a finding seen in older adults.^30^ While engagement in such activities is positive, it is concerning that there was little mention of resistance exercise; potentially highlighting a gap in participants’ knowledge and understanding of the importance and utility of resistance exercise. While several participants did mention the use of a gym for exercise, interestingly, those who specifically discussed muscle strengthening and/or resistance exercise were all exclusively male, suggesting a potential underlying gender difference. Gender-specific barriers to exercise are well reported in the literature;^31 32^ however, it is important to note that this was not a specific focus of this study and thus was not probed further. Therefore, future qualitative endeavours should further explore gender-specific barriers for resistance exercise in SA populations. While our finding is from a limited number of participants, larger population studies have shown that engagement in regular strength training (ie, two times per week) is lower in Asian women compared with men (33% vs 43%)^10^; suggesting a need to investigate this difference thoroughly.

Research has shown that relationships, knowledge, beliefs and environmental concerns act as barriers to PA in adult SA populations.^21^ Here, the most commonly cited barriers were participants’ lifestyles, their work and demands on their time. Those in full-time work expressed more concern about the lack of flexibility, in addition to a lack of motivation; which mirrors that of other lifestyle interventions within SA populations.^33^ Further, while not a significant focus, specific cultural concerns were raised as barriers, and interestingly, the majority of such factors were raised by women. One such barrier was the time commitment associated with the role SA women often play in the household in regard to chores and food preparation. Furthermore, female participants vocalised that some may feel too uncomfortable in mixed-sex gyms, although this may not be specific to SA women, as this is a regularly cited barrier for all women.^34^ Nonetheless, culturally adapted PA interventions in Middle Eastern women have proved successful in overcoming similar religious and cultural considerations (eg, women’s only gyms)^35^ and thus should be considered in SA women.

Participants also shared motivators and facilitators that could increase exercise engagement. Interestingly, it appears these factors did not appear to be culturally specific and are commonly seen across a range of populations; for example, participants cited social support, enjoyment, progress recognition, incorporating activity into daily routines and purposeful scheduling. One over-riding theme was the concept of receiving sufficient peer support and engaging in grouped activities; in turn, it was suggested that this would enhance enjoyment and create a sense of joint venture. It is therefore evident that future endeavours and/or interventions in SA adults should consider employing such approaches to enhance engagement and adherence.

Participants stated the need for more education on different modes of exercise and their health benefits, with a particular emphasis on a limited understanding of muscle strength. This knowledge gap may be due to the lack of awareness of the UK PA guidelines which state the need for two resistance exercise sessions per week, as only 2/15 participants (13%) were aware of these guidelines; of which both participants worked within the NHS. Participants stated they largely obtained exercise knowledge from social media, family and gym trainers. Alarmingly, health misinformation is prevalent on social media^36^ and so new routes must be sought to disseminate accurate, evidence-based knowledge on resistance exercise into the SA public. Considerations may also be required to address the linguistic diversity that is present in SA populations which may in part drive this knowledge gap. Indeed, previous research has highlighted that the most commonly successful surface structure cultural adaptation to increase PA is language adjustments, including bilingual staff/resources or exclusively delivering sessions or materials in the language of participants.^37 38^

There are inherently a few limitations within this study which must be acknowledged. First, while endeavours to ensure an equal sex split and a varied representation from each major religion were successful, we acknowledge shortcomings in socioeconomic representation. Indeed, participants here were largely well educated (9/15 university level) and in professional job settings. Therefore, we note an under-representation of individuals from lower income and/or lower education backgrounds, and therefore future work should specifically target these socioeconomic populations.

## Conclusion

Our findings reveal that while SA adults recognise the importance of PA and exercise, there is limited awareness of specific guidelines and a gap in understanding different forms of exercise, such as resistance exercise. To enhance the nation’s health, particularly by promoting exercise among ethnic minority populations, timely and collaborative action is essential. As such, this study provides preliminary research and recommendations for further research in this area.

Key stakeholders, including community leaders, academic researchers, healthcare professionals, employers and local councils must work together to drive meaningful change. Initiatives such as community walking groups, exercise buddy systems and resistance training sessions can provide practical solutions. Additionally, addressing linguistic diversity within ethnic minority groups requires innovative approaches to disseminating information. These might include discussing PA in places of worship, developing culturally relevant guidance and leveraging reputable social media platforms. Employers also have a role in fostering well-being by offering flexibility and supporting initiatives that encourage active and healthy lifestyles, benefiting both individuals and the broader community.

## Data Availability

Data are available upon reasonable request. All data relevant to the study are included in the article or uploaded as supplementary information.
