# Supplementary material for: Exploring barriers, motivators and facilitators for physical activity and exercise in a UK South Asian community: a qualitative study
Source: BMJ Open. 2025 Feb 19;15(2):e097268. doi: 10.1136/bmjopen-2024-097268 (PMC11840903; doi:10.1136/bmjopen-2024-097268)
Supplement: Supplementary file 1 [file bmjopen-2024-097268.reviewer_comments.pdf]

PEER REVIEW HISTORY

BMJ Open publishes all reviews undertaken for accepted manuscripts. Reviewers are asked to complete a checklist review form and are provided with free text boxes to elaborate on their assessment. These free text comments are reproduced below.

This paper was submitted to another journal from BMJ but declined for publication following peer review. The authors addressed the reviewers' comments and submitted the revised paper to BMJ Open. The paper was subsequently accepted for publication at BMJ Open.

ARTICLE DETAILS

Title (Provisional)

Exploring barriers, motivators and facilitators for physical activity and exercise in a UK South Asian Community: a qualitative study.

Authors

Deshmukh, Ashwini; Kumar, Kanta; Quinlan, Jonathan

VERSION 1 - REVIEW

**Reviewer**	**1**
**Name**	**Reviewer 1**
**Affiliation**	
**Date**	**27-Sep-2024**
**COI**	**None**

Comments not published pending reviewer's consent

**Reviewer**	**2**
**Name**	**Mahmood, Bushra**
**Affiliation**	**University of British Columbia**
**Date**	**27-Sep-2024**
**COI**	**None**

I would like to thank all authors for their commitment towards their qualitative study. I have some comments.

Abstract:
Methods:
Line 23 – take our mean age and sex info – belongs in the results section.

Main Manuscript

Introduction:
Line 15 – add info on 150-300 min of moderate intensity of PA (under WHO guidelines) as for many of us, achieving moderate PA is relatively easier than vigorous PA.
Line 36 : consider restructuring the sentence
Second para: specify whether the PA levels were self-reported or measured objectively.
Line 47: consider replacing ‘evoke’ with another word
Line 52: specify some ‘environmental’ concerns to give readers an idea about what you are referring to.
General: how do you define PA and how do you differentiate between PA and exercise?
Line31: Consider restructuring sentence- also mention if public partners were given approval to review confidential data and if participants were made aware of this.

Methods:
Line 18 – elucidate the various constructs of SDT and how they were captured or measured in your study
Results;
Line 12: is this a reference to NHS Pa guidelines or dietary guidelines ?

Discussion:
Line 38 : Not sure if ‘diabetes’ is the focus – there is little or no mention of it earlier in the text
Line 39-41: not sure if this study is about ‘intervention’ or any ‘intervention measures’ were ‘proactively’ discussed in depth with the participants. Not sure why the word ‘mimic’ is used here.
Line 50: please explain which socio-economic group was not represented and how that may have biased your results.
The discussion feels a little thin on reference to existing literature in some places – I would suggest the authors look up literature on culturally adapting PA interventions and see if they align with some of the conclusions of this study.
Overall, it would be helpful to get this proof-read as there are a significant number of grammatical errors.

**Reviewer**	**3**
**Name**	**Reviewer 3**

**Affiliation**	
**Date**	**27-Sep-2024**
**COI**	**None**

Comments not published pending reviewer's consent

**Reviewer**	**4**
**Name**	**Ampiah, Paapa Kwesi**
**Affiliation**	
**Date**	**27-Sep-2024**
**COI**	**None**

Main comment
Well done on putting together this manuscript. Overall, it provides essential knowledge to existing literature and you provide a clear direction for future research. However, I'm concerned you do not stay within the remit of qualitative studies at certain points and appear to overstate the impact/relevance of the results. Its important to stay within the exploratory nature of qualitative research and not attempt to generalize findings or make inferences without caution. Your methods section will need a major update to present better rigor, transparency and audit trail of your study. Its important to demonstrate the conceptual underpinnings in your methods section. Your discussion should stay within the remit of qualitative studies all the time.
Also, consider proofreading again and write in more scientific language throughout the manuscript. Consider the structure of your write-up as well in terms of paragraphing and coherence.

Abstract
Introduction
Your introduction is good. However, its important to contextualize the issues you highlight (that is, reduced mortality, low engagement with PA and strengthening exercises.). Its importat5nt to briefly demonstrate which contexts you report the evidence; whether globally, continentally, nationally or locally. This will support your rationale for your study and study setting.

Methods
You need to provide where you recruited your participants from in this section. Also, did you predetermine that all your interviews will last one hour, was there a range, or all interviews ended up being one hour? It will be important to demonstrate how the self-determination theory was used to inform your interview guide in the main body of

your manuscript.

Results
Consider using a more scientific word to describe nuanced.

Conclusion
Although your results in the abstract may be a reflection of the study, there appears to be some lack of coherence with the the results section. Please ensure a logical flow in the various subsections of the abstract to make it more coherent.

Introduction
Line 7 - 11: Please rewrite for clarity. Do you mean muscle mass and muscle strength are interventions?

Materials and methods
Line 11 and 12: This statement needs to be referenced
Line 12 - 15: Please use more scientific language in drafting your manuscript.
line 19 -21: this is strong enough. I suggest you provide a more robust justification for the choice and explain clearly how it 'elucidates the motivation behind exercise'

Participant recruitment
Line 32 - 35: You need to be clear why you used a sample of 15 participants. Also, the places you recruited your participants from, which cities/communities were they located? You need to provide some rationale for your choice of recruitment centres. Also, please be clear about what sampling method you applied in your study.
Line 37 - 39: Did you have an age limit for your participants? If yes, you need to provide this and the rationale.

Interview development and process
Line 45 - 46: Please clearly demonstrate how the SDT informed the interview guide; this is important for transparency.
Line: 3 - 6: How did you ensure that the translated text represented exactly what was translated from the source document? If there was any back translation, please indicate this. If not, please indicate why back translation was not necessary to ensure rigor.

Thematic analysis
Line 11 - 14: what evidence/literature informed your choice of thematic analysis? Please be clear on this.
Line 15 - 17: Please indicate who were involved in the discussions and the role they played. Were they involved in the coding, developing categories and themes? Was there a peer review process? ....and how was this operationalised?

Patient and public involvement
Line 30 - 34: You need to be transparent about who these public partners were, their role in the design of the interview guide (especially because you have already mentioned the patient collaborator earlier) and what their level of involvement with the analysis and interpretation was, in relation to their expertise. There needs to be transparency in your reportage.

Results
You results are very clear and presented in a very coherent manner. Well done!

Discussion
Line 7 - 9: This statement needs to be referenced.
Line 8 - 14: Since your study is a qualitative study, its important to stay within the remit. The attempt to discuss the lack of knowledge of women should be contextualised within the remit of qualitative studies so as not to appear as though you are generalising your findings. This should be clearly stated and perhaps suggest further exploration to establish this evidence.
Line 22 - 25: Please discuss this within the remit of qualitative studies so that you do not overstate the impact/implications of your results.

You have to provide a logical flow to the thematic analysis process and demonstrate who was involved in what for transparency.

The reviewer provided a marked copy with additional comments. Please contact the publisher for full details.

**Reviewer**	**5**
**Name**	**Reviewer 5**
**Affiliation**	
**Date**	**27-Sep-2024**
**COI**	**None**

Comments not published pending reviewer's consent

VERSION 1 - AUTHOR RESPONSE

**Reviewer comment**	Response
**Reviewer 2:**	
Intro Line 23 – take our mean age and sex info – belongs in the results section.	We thank the reviewer for this comment , this has now been removed.
Intro Line 15 – add info on 150-300 min of moderate intensity of PA (under	We thank the reviewer for this comment , this information has now been added.

WHO guidelines) as for many of us, achieving moderate PA is relatively easier than vigorous PA.	
Intro Line 36 : consider restructuring the sentence	We thank the reviewer for this comment , this sentence has now been condensed and rewritten.
Intro Second para: specify whether the PA levels were self-reported or measured objectively.	We thank the reviewer for this comment, this detail has now been added to reflect PA levels measured objectively within the references provided.
Intro Line 47: consider replacing ‘evoke’ with another word	This word has now been replaced
Intro Line 52: specify some ‘environmental’ concerns to give readers an idea about what you are referring to.<br>General: how do you define PA and how do you differentiate between PA and exercise?	We thank the reviewer for this comment, an example of environmental concerns has now been provided in text. In addition, wording has been added to the introduction to differentiate between PA and exercise.
Methods Line31: Consider restructuring sentence- also mention if public partners were given approval to review confidential data and if participants were made aware of this	We thank the reviewer for this comment, this sentence has now been amended and details around public partner consent is now provided within the PPI section.
Methods Line 18 – elucidate the various constructs of SDT and how they were captured or measured in your study	The following text has now been added: “DT is a motivational framework that has been widely applied across various aspects of life, including health. Health is an intrinsic goal for everyone, profoundly shaped by personal habits and lifestyle choices. Motivation, defined as the energy directed towards achieving a goal, plays a crucial role in influencing these choices and sustaining the necessary changes to maintain good health. According to SDT, three fundamental psychological needs underpin motivation: autonomy (the sense of willingly choosing and endorsing one’s actions), competence (the feeling of mastery and effectiveness in one’s activities), and relatedness (the need to feel connected to and supported by others). These needs collectively drive our capacity for sustained behavioural change and personal growth (Eassey et al., 2020).”
Results Line 12: is this a reference to NHS Pa guidelines or dietary guidelines ?	We thank the reviewer for this comment, this has now been clarified, this is in reference to PA guidelines.

Discussion, Line 38 : Not sure if 'diabetes' is the focus – there is little or no mention of it earlier in the text –	We agree that the focus of this paper was not on diabetes, and as such we have not provided significant context within introduction. We present the views of the general public, some of who did have some form of comorbidity and as such their opinions in some cases focussed on their condition.
Discussion, Line 39-41: not sure if this study is about 'intervention' or any 'intervention measures' were 'proactively' discussed in depth with the participants. Not sure why the word 'mimic' is used here. –	We thank the reviewer for this comment , this has now been reworded so that it does not imply an intervention was used or discussed, but instead that the participants typically mentioned low intensity exercise modes. The word mimic has been replaced.
Discussion, Line 50: please explain which socio-economic group was not represented and how that may have biased your results. – lower income, lower education, socio-economical backgrounds	Details have now been added as to which groups were underrepresented in this study. In addition, we have removed the inference that our findings may in fact be worse in the general population when all socio-economic groups are considered.
The discussion feels a little thin on reference to existing literature in some places – I would suggest the authors look up literature on culturally adapting PA interventions and see if they align with some of the conclusions of this study.	We thank the reviewer for this suggestion. Additional references which focus upon culturally adapted interventions have now been added into the discussion, including:<br><br>Siddiqui, F., Koivula, R. W., Kurbasic, A., Lindblad, U., Nilsson, P. M., & Bennet, L. (2018). Physical Activity in a Randomized Culturally Adapted Lifestyle Intervention. American Journal of Preventive Medicine, 55(2), 187–196. https://doi.org/10.1016/j.amepre.2018.04.016<br><br>Dennaoui, N., Guagliano, J. M., Kolt, G. S., Jammal, M., & George, E. S. (2024). A systematic review of culturally adapted physical activity interventions for culturally and linguistically diverse children and adolescents. In Preventive Medicine (Vol. 183). Academic Press Inc. https://doi.org/10.1016/j.ypmed.2024.107979<br><br>Montayre, J., Neville, S., Dunn, I., Shrestha-Ranjit, J., & Wright-St. Clair, V. (2020). What makes community-based physical activity programs for culturally and linguistically diverse older adults effective? A systematic review. In Australasian Journal on Ageing (Vol.

	39, Issue 4, pp. 331–340). Blackwell Publishing. https://doi.org/10.1111/ajag.12815
Overall, it would be helpful to get this proof-read as there are a significant number of grammatical errors.	The manuscript has now been proof-read by an additional academic colleague and rewritten in many areas to improve clarity and succinctness.
**Reviewer 4 (In document comments)**	
Abstract<br>Introduction<br>Your introduction is good. However, its important to contextualize the issues you highlight (that is, reduced mortality, low engagement with PA and strengthening exercises.). Its importat5nt to briefly demonstrate which contexts you report the evidence; whether globally, continentally, nationally or locally. This will support your rationale for your study and study setting.	We have now clarified that this is within a UK South Asian population for context within the abstract, and provide further context within the methods section (i.e., the local Birmingham South Asian community).
Abstract<br>Methods<br>You need to provide where you recruited your participants from in this section. Also, did you predetermine that all your interviews will last one hour, was there a range, or all interviews ended up being one hour? It will be important to demonstrate how the self-determination theory was used to inform your interview guide in the main body of your manuscript.	Details have been added as to where participants were recruited from. Interviews were scheduled for a total of one hour allowing time for the consent process. Based upon previous experience this was sufficient time for the number of questions and interviews were typically completed in 45-55minutes. As such, we have removed this detail from the abstract to avoid confusion.
Abstract<br>Results<br>Consider using a more scientific word to describe nuanced.	This word has been replaced.
Abstract<br>Conclusion<br>Although your results in the abstract may be a reflection of the study, there appears to be some lack of coherence with the the results section. Please ensure a logical flow in the various subsections of the abstract to make it more coherent.	We thank the reviewer for this comment, the conclusion has been rewritten so that it flows more logically.

Introduction<br>Line 7 - 11: Please rewrite for clarity. Do you mean muscle mass and muscle strength are interventions?	We thank the reviewer for this comment, we recognise that the wording would imply they are interventions themselves. However, it was meant that reductions in muscle mass and strength are points of where intervention is needed i.e., targeting declines in muscle health. This has since been reworded for clarity.
Materials and methods<br>Line 11 and 12: This statement needs to be referenced	Reference is now provided.
Materials and methods<br>Line 12 - 15: Please use more scientific language in drafting your manuscript.	Wording has now been amended.
Materials and methods<br>line 19 -21: this is strong enough. I suggest you provide a more robust justification for the choice and explain clearly how it 'elucidates the motivation behind exercise'	We thank the reviewer for this comment, the following text has now been added: "DT is a motivational framework that has been widely applied across various aspects of life, including health. Health is an intrinsic goal for everyone, profoundly shaped by personal habits and lifestyle choices. Motivation, defined as the energy directed towards achieving a goal, plays a crucial role in influencing these choices and sustaining the necessary changes to maintain good health. According to SDT, three fundamental psychological needs underpin motivation: autonomy (the sense of willingly choosing and endorsing one's actions), competence (the feeling of mastery and effectiveness in one's activities), and relatedness (the need to feel connected to and supported by others). These needs collectively drive our capacity for sustained behavioural change and personal growth (Eassey et al., 2020)."
Participant recruitment<br>Line 32 - 35: You need to be clear why you used a sample of 15 participants. Also, the places you recruited your participants from, which cities/communities were they located? You need to provide some rationale for your choice of recruitment centres. Also, please be clear about what sampling method you applied in your study.	The inclusion of only 15 interviews is deemed sufficient and informed by other qualitative publications. We herein focussed on depth of understanding rather than quantity: Malterud K, Siersma VD, Guassora AD. Sample Size in Qualitative Interview Studies: Guided by Information Power. Qual Health Res. 2016;26(13):1753-60. As such the following text was added: "The recruitment of 15 participants was deemed sufficient as informed by previous literature (Malterud et al., 2016)"

	We have now specified that recruitment occurred in the city of Birmingham, in addition to providing rationale for the choice of recruitment sites. Further, details are provided as to the method of sampling.
Participant recruitment<br>Line 37 - 39: Did you have an age limit for your participants? If yes, you need to provide this and the rationale.	There was no age limit provided for this study as we wished to explore the views across the lifespan.
Interview development and process<br>Line 45 - 46: Please clearly demonstrate how the SDT informed the interview guide; this is important for transparency.	The following text has been added:<br>"The questions were very sepeiclally geared around the three fundamental psychological needs underpin motivation: autonomy (the sense of willingly choosing and endorsing one's actions), competence (the feeling of mastery and effectiveness in one's activities), and relatedness (the need to feel connected to and supported by others) (Eassey et al., 2020)."
Interview development and process<br>Line: 3 - 6: How did you ensure that the translated text represented exactly what was translated from the source document? If there was any back translation, please indicate this. If not, please indicate why back translation was not necessary to ensure rigor.	The following text has now been added:<br>"Interview transcription was consequently completed by an independent professional company, with interviews completed in Hindi translated into English. Using the MAPI guidelines (Acquadro, 2012), the interviews were directly translated from Hindi into English from audio recording. We did not complete any specific back translation as the translated script was read by the research associate who was themselves fluent in Hindi, and these were checked against the audio recording for accuracy."
Thematic analysis<br>Line 11 - 14: what evidence/literature informed your choice of thematic analysis? Please be clear on this.	The following text has now been added:<br>"…informed by Braun and Clarke (Braun & Clarke, 2014, 2019)."
Thematic analysis<br>Line 15 - 17: Please indicate who were involved in the discussions and the role they played. Were they involved in the coding, developing categories and themes? Was there a peer review process? ....and how was this operationalised?	We thank the reviewer for this comment, this is now corrected on page 5 with the addition of the following text: "A meeting took place with our public partner to gain their agreement on the overall merging themes and any discrepancies were corrected after the meeting as mentioned below."
Patient and public involvement<br>Line 30 - 34: You need to be transparent about who these public	The following text has now been inserted:<br>"Participant consent was obtained to allow public partner to review interview text,

partners were, their role in the design of the interview guide (especially because you have already mentioned the patient collaborator earlier) and what their level of involvement with the analysis and interpretation was, in relation to their expertise. There needs to be transparency in your reportage.	however all identifiable data was removed from transcripts and only identified by unique study code. All public partners were provided renumeration for their time and contribution."
Discussion<br>Line 7 - 9: This statement needs to be referenced.	Appropriate reference has now been added.
Discussion<br>Line 8 - 14: Since your study is a qualitative study, its important to stay within the remit. The attempt to discuss the lack of knowledge of women should be contextualised within the remit of qualitative studies so as not to appear as though you are generalising your findings. This should be clearly stated and perhaps suggest further exploration to establish this evidence.	We acknowledge that it is difficult to make generalised statements from a small sample size and we have amended the wording to reflect this. Further, we have removed the discussion around quantitative data linking women's health and strengthening exercise.
Discussion<br>Line 22 - 25: Please discuss this within the remit of qualitative studies so that you do not overstate the impact/implications of your results.	This comment has now been addressed with additional reference to qualitative studies.

VERSION 2 - REVIEW

**Reviewer**	**4**
**Name**	**Ampiah, Paapa Kwesi**
**Affiliation**	
**Date**	**31-Dec-2024**
**COI**	**None**

Thank you for the opportunity to review this manuscript. Overall, the authors have made a relevant addition to literature in a very robust way and they should be commended. I just have a few comments for potential correction and need clarifications on a few points also. Please see below.

Abstract

Well done on the abstract. Your background, methods, results and conclusion are clear. You will need to provide robust evidence for strengthening exercises and their relevance to 'independence, quality of life and healthy lifespan' in your main text.

What this study adds

Since this is a qualitative study, its important to use terminology that do not depict making quantitative inferences. My recommendation will be to change words like 'Significant' and replace them with words relevant to qualitative research or delete them entirely.

How this study might affect research, practice, or policy

At best this qualitative study provides preliminary knowledge to your research question. Hence, your recommendation should be made with that view of your study providing preliminary knowledge and a recommendation for further research in this area. Secondly, making recommendations for emphasis on PA engagement in SA communities and 'new routes of relevant culturally relevant information dissemination' should be made with reference to and within the remit of your qualitative study, so as not to draw conclusions that are not borne out of your study.

Introduction

Line 17 to 20 Please provide a reference for the definitions

Line 23 - Please write minutes in full

You introduction is written in very coherent and compelling manner.

Materials and methods

There is a good focus on theory; please consider the following.

Your data management and collection processes to ensure confidentiality, participant autonomy, rigor in the data collection and management processes, and how researchers approached this study (your stance on the research topic) should be outlined explicitly.

Results

line 34 Spelling of physical

line 45 Was it your expectation that participants will specifically be able to identify/acknowledge resistance exercise; did your research team take the position that resistance exercise should be mentioned by participants or you had certain key words that related to same? I think you should clarify what the expectation of participants in relation resistance exercises was. Did you expect commentary around exercises that strengthen muscles, or your focus was on the words 'resistive exercise'?

line 41 - 43 was it the expectation that participants will be able to identify different types of exercise? What was the rationale for this? And how does this answer your research question?

Discussion

Page 11 line 20 for proactive?

I thought your study sought to explore the barriers SA participant had towards PA and exercise? I wonder why you say gender barriers and cultural concerns were not a focus of your study. Did you have a specific focus other than these?

Are there any methodological strengths or limitations you want to highlight? What is the expertise of the research team in conducting qualitative research?

Conclusion line 50 Please avoid terminologies like significant.

The reviewer provided a marked copy with additional comments. Please contact the publisher for full details.

**Reviewer**	**2**
**Name**	**Mahmood, Bushra**
**Affiliation**	**University of British Columbia**
**Date**	**10-Jan-2025**
**COI**	**None**

Thank you for focusing your research on an important public health issue of low rates of PA among SA ethnic minority group. This is a pattern that is seen in many other western countries where South Asian immigrants have settled and is of concern because of the association of PA with chronic disease.

Findings regarding limited knowledge of SA about resistance exercise or muscle building is relatively new as few researchers have looked into this aspect of PA before. I would suggest that you put a little more emphasis on this in your manuscript. Also, it is not clear whether it was your intention to explore this aspect or was it a chance finding? If it was the focus, I do not see any specific question in the guide that is directed at exploring the knowledge level regarding resistance exercise.

Lastly, manuscript would benefit from editing for grammatical errors and restructuring of sentences.

Page 3- Line 23: which recommends adults engage in 75 to 150 minutes of vigorous-intensity or 150-300 min of moderate intensity aerobic physical activity; (specify this is per week)

Page 5- 34: classified – instead of 'classed'

Page 3- 46-50: not sure what the proportion of SA population has to with opportunities for intervention? Perhaps a better association would be with the cost to healthcare system or NHS of 9.3% or 5 million people with suboptimal levels of PA and high risk of non-communicable disease.

Page 5 : Line 40:: the research associate who was themselves fluent in Hindi – grammatical error

P6:56: in their diaries. – not sure what this means

P8-53: e it wasn't too expensive." Add in brackets [ ] what 'it' is referring to . Gym?

P9-line 11: "Asian social media" – is this South Asian or Asian? Clarify as two are different.

P11 Line 23: 'identified and were observed that engagement in resistance exercise was linked to a lack of specific knowledge.' Need to re-word this sentence

---

VERSION 2 - AUTHOR RESPONSE

Reviewer: 4
Dr. Paapa Kwesi Ampiah
Comments to the Author:
Thank you for the opportunity to review this manuscript. Overall, the authors have made a relevant addition to literature in a very robust way and they should be commended. I just have a few comments for potential correction and need clarifications on a few points also. Please see below.
Response: Many thanks for your time and efforts in completing this peer review and providing constructive suggestions. We have provided responses to each of your comments below with line references.

Abstract
Well done on the abstract. Your background, methods, results and conclusion are clear. You will need to provide robust evidence for strengthening exercises and their relevance to 'independence, quality of life and healthy lifespan' in your main text.
Response: Thank for you your kind words. We have provided additional references to strengthen this point in the introduction on line 237

What this study adds
Since this is a qualitative study, its important to use terminology that do not depict making quantitative inferences. My recommendation will be to change words like 'Significant' and replace them with words relevant to qualitative research or delete them entirely.
Response: These sections have now been removed following request from the Editor and the journal format. As such, no other specific changes were made in response to this. However, we have made efforts to remove the term 'significant' throughout this paper.

How this study might affect research, practice, or policy
At best this qualitative study provides preliminary knowledge to your research question. Hence, your recommendation should be made with that view of your study providing preliminary knowledge and a recommendation for further research in this area. Secondly, making recommendations for emphasis on PA engagement in SA communities and 'new routes of relevant culturally relevant information dissemination' should be made with reference to and within the remit of your qualitative study, so as not to draw conclusions that are not borne out of your study.
Response: Similarly, as above, this section has now been removed on request of the Editor and the journal format.

Introduction

Line 17 to 20 Please provide a reference for the definitions

Response: Reference has now been provided

Line 23 - Please write minutes in full

Response: This change has now been made

You introduction is written in very coherent and compelling manner.

Thank you for your comment

Materials and methods

There is a good focus on theory; please consider the following.

Your data management and collection processes to ensure confidentiality, participant autonomy, rigor in the data collection and management processes, and how researchers approached this study (your stance on the research topic) should be outlined explicitly.

Response: Many thanks for your suggestion. We have added a new section on data management to the methods section to provide further details. Lines 341-348

Results

line 34 Spelling of physical

Response: This has now been corrected

line 45 Was it your expectation that participants will specifically be able to identify/acknowledge resistance exercise; did your research team take the position that resistance exercise should be mentioned by participants or you had certain key words that related to same? I think you should clarify what the expectation of participants in relation resistance exercises was. Did you expect commentary around exercises that strengthen muscles, or your focus was on the words 'resistive exercise'?

Response: Thank you for your comment. The interview guide was constructed in order to explore the understanding of physical activity and exercise as concepts, with resistance exercise or strengthening exercise forming one part of this. We did not focus on specific terminology, as we know that members of the public may not be aware of these terms, nor whether certain exercises would be classed as muscle strengthening or as resistance exercise. Instead, if participants discussed exercises that fit the criteria, then this was noted (e.g., I like to use the leg press at the gym, or I use the weight machines at my gym). Importantly, if participants mentioned the use of a gym, then the researcher would probe as to what equipment used was, or what exercises were performed.

line 41 - 43 was it the expectation that participants will be able to identify different types of exercise? What was the rationale for this? And how does this answer your research question?

Response: Thank you for this comment, it raises another important issue. The rationale of this vignette was to explore participant understanding of the role muscle strength plays in activities of daily living and maintaining one's independence, i.e., carrying shopping or climbing stairs. These concepts are robust in the literature, and regularly discussed concepts with this field of research, however, there is often a disconnect within the general public. In this instance, it was apparent from our findings that participants still maintained a focus on cardiovascular exercise which highlighted a lack of consideration or awareness of muscle strength and thus strengthening exercise to overcome those challenges. This lack of awareness in itself identifies a key barrier to engagement in strengthening/resistance exercise and as such answered a component of the first research question.

Discussion

Page 11 line 20 for proactive?

Response: This change has now been made

I thought your study sought to explore the barriers SA participant had towards PA and exercise? I wonder why you say gender barriers and cultural concerns were not a focus of your study. Did you have a specific focus other than these?
Response: Thank you for your comment. You are correct, the study did seek to explore the barriers to exercise and PA in South Asian adults. We purposely chose not to have a direct line of questioning relating to the role of gender or cultural concerns, as we wanted participant responses to occur organically. The questions were open-ended, and the direction of the discussion was led by the participant. In the case that the participants discussed either of these concepts, then this was explored in greater depth by the researcher.

Are there any methodological strengths or limitations you want to highlight? What is the expertise of the research team in conducting qualitative research?
Response: The methodological strengths and limitations are now added to the manuscript following the abstract, lines 36-44 as per the Editors request and journal format. Dr Kanta Kumar has over two decades of experience in qualitative research with a specific focus on musculoskeletal conditions (e.g., rheumatoid arthritis) and the role of ethnicity (largely South Asian populations). Dr Ashwini Deshmukh has completed numerous qualitative research studies under the supervision of Dr Kanta Kumar. Finally, whilst limited experience as a qualitative researcher, Dr Jonathan Quinlan has extensive experience in muscle health, sarcopenia, and frailty in respect to age, disease and ethnicity.

Conclusion line 50 Please avoid terminologies like significant.

Response: This language has now been removed in this instance, but also throughout the manuscript.

Reviewer: 2
Dr. Bushra Mahmood, University of British Columbia
Comments to the Author:
Thank you for focusing your research on an important public health issue of low rates of PA among SA ethnic minority group. This is a pattern that is seen in many other western countries where South Asian immigrants have settled and is of concern because of the association of PA with chronic disease.
Response: Thank you for completing this review of our manuscript and for the comments and suggestions listed below. We agree that this is a key problem to tackle. Our response to each question/comment is provided underneath.

Findings regarding limited knowledge of SA about resistance exercise or muscle building is relatively new as few researchers have looked into this aspect of PA before. I would suggest that you put a little more emphasis on this in your manuscript. Also, it is not clear whether it was your intention to explore this aspect or was it a chance finding? If it was the focus, I do not see any specific question in the guide that is directed at exploring the knowledge level regarding resistance exercise.
Response: Thank you for your comment. This finding was a combination of the two factors you mention above. In the first instance, vignette one was specifically used to explore the understanding of the important role muscle strength plays in activities of daily living such as carrying shopping or climbing stairs. Inherently, one way to improve muscle strength is through resistance exercise/muscle strengthening activities. Secondly, within the interviews, the participants were asked to discuss the difference between exercise and physical activity, in addition to any forms of exercise that they engaged in. As such, we did not directly ask them about resistance exercise, but if they mentioned the

use of the gym, the researcher did follow up to understand if any strengthening exercises were completed.

Lastly, manuscript would benefit from editing for grammatical errors and restructuring of sentences.
Response: In addition to our own editing, this manuscript has now also been proofread by a senior colleague and various grammatical changes have been made throughout the manuscript.

I have some other minor revisions suggested in the attached document.

Page 3- Line 23: which recommends adults engage in 75 to 150 minutes of vigorous-intensity or 150-300 min of moderate intensity aerobic physical activity; (specify this is per week).
Response: This detail has now been added.

Page 5- 34: classified – instead of 'classed'
Response: Thank you for highlighting this. This has now been amended.

Page 3- 46-50: not sure what the proportion of SA population has to with opportunities for intervention? Perhaps a better association would be with the cost to healthcare system or NHS of 9.3% or 5 million people with suboptimal levels of PA and high risk of non-communicable disease.
Response: This data is cited to demonstrated that South Asian adults comprise a considerable proportion of the UK population. Further, the current state of the literature has not thoroughly considered whether different approaches may be needed depending on one's ethnicity, which in turn would limit the applicability of existing public health interventions.

Page 5 : Line 40:: the research associate who was themselves fluent in Hindi – grammatical error
Response: Thank you for highlighting this. This word has now been removed.

P6:56: in their diaries. – not sure what this means
Response: We have clarified this by altering the wording to read 'flexibility in their working diaries'.

P8-53: e it wasn't too expensive." Add in brackets [ ] what 'it' is referring to . Gym? P9-line 11: "Asian social media" – is this South Asian or Asian? Clarify as two are different.
Response: Thank you for highlighting this. This has now been added on lines 475, and the distinction between South Asian and Asian has now been made.

P11 Line 23: 'identified and were observed that engagement in resistance exercise was linked to a lack of specific knowledge.' Need to re-word this sentence
Response: Thank you for highlighting this. This sentence has now been corrected, we noticed this was a typing error.
